# Comparative Proteomics Indicates That Redox Homeostasis Is Involved in High- and Low-Temperature Stress Tolerance in a Novel Wucai (*Brassica campestris* L.) Genotype

**DOI:** 10.3390/ijms20153760

**Published:** 2019-08-01

**Authors:** Lingyun Yuan, Jie Wang, Shilei Xie, Mengru Zhao, Libing Nie, Yushan Zheng, Shidong Zhu, Jinfeng Hou, Guohu Chen, Chenggang Wang

**Affiliations:** 1 College of Horticulture, Vegetable Genetics and Breeding Laboratory, Anhui Agricultural University, 130 West Changjiang Road, Hefei 230036, China; 2 Provincial Engineering Laboratory for Horticultural Crop Breeding of Anhui, 130 West of Changjiang Road, Hefei 230036, China; 3 Department of vegetable culture and breeding, Wanjiang Vegetable Industrial Technology Institute, Maanshan 238200, China

**Keywords:** wucai, low-temperature stress, high-temperature stress, proteomics, redox homeostasis, GLU1, glutathione

## Abstract

The genotype WS-1, previously identified from novel wucai germplasm, is tolerant to both low-temperature (LT) and high-temperature (HT) stress. However, it is unclear which signal transduction pathway or acclimation mechanisms are involved in the temperature-stress response. In this study, we used the proteomic method of tandem mass tag (TMT) coupled with liquid chromatography-mass spectrometry (LC-MS/MS) to identify 1022 differentially expressed proteins (DEPs) common to WS-1, treated with either LT or HT. Among these 1022 DEPs, 172 were upregulated in response to both LT and HT, 324 were downregulated in response to both LT and HT, and 526 were upregulated in response to one temperature stress and downregulated in response to the other. To illustrate the common regulatory pathway in WS-1, 172 upregulated DEPs were further analyzed. The redox homeostasis, photosynthesis, carbohydrate metabolism, heat-shockprotein, and chaperones and signal transduction pathways were identified to be associated with temperature stress tolerance in wucai. In addition, *35S:BcccrGLU1* overexpressed in *Arabidopsis,* exhibited higher reduced glutathione (GSH) content and reduced glutathione/oxidized glutathione (GSH/GSSG) ratio and less oxidative damage under temperature stress. This result is consistent with the dynamic regulation of the relevant proteins involved in redox homeostasis. These data demonstrate that maintaining redox homeostasis is an important common regulatory pathway for tolerance to temperature stress in novel wucai germplasm.

## 1. Introduction

As a consequence of climate change, temperature stress is becoming a major concern in plant research. Changes in low temperature (LT) and high temperature (HT) are expected to greatly affect enzyme activities, photosynthesis, carbon assimilation, DNA/RNA stability, membrane fluidity, and transcription and translation rates [1]. Exposure to extreme temperatures usually reduces photosynthesis efficiency, and the reduced photosynthesis disturbs cellular homeostasis and promotes lipid peroxidation, either by increasing the production of reactive oxygen species (ROS) or by decreasing the scavenging of superoxide anion (O_2_^•−^) in the cell [2]. The accumulation of ROS depends on changes in the redox state of cells, and thus it could function to reset the redox state and maintain redox homeostasis [3]. Because the sulfur-containing amino acids cysteine and methionine are sensitive to redox potential, changes in the redox state can affect protein structure and folding [4]. Changes in redox potential may also alter enzyme activity, biochemical reactions, and plant physiological processes, which can negatively affect plant survival [5].

Wucai (*Brassica campestris* L. ssp. *chinensis* var. *rosularis* Tsen et Lee.), a species of non-heading Chinese cabbage (NHCC) and one of the most important leafy vegetables in China, is now extensively cultivated worldwide. Injury from temperature stress can decrease wucai yield and edible quality [6]. The novel genotype WS-1, which was previously identified from a series of wucai germplasms, exhibited higher tolerance to LT and HT than other genotypes used in previous experiments. Under HT, WS-1 had a greater net photosynthesis rate, antioxidative capacity, and carbon–nitrogen assimilation efficiency than other genotypes [6,7,8]. Under LT, the malondialdehyde (MDA) content and relative electric conductivity were lower in WS-1 than in other genotypes, while levels of soluble sugar, free proline, and chlorophyll were higher than in others [9]. However, it is unclear which regulatory pathway plays a dominant role in responding to thermal stress, and whether LT and HT tolerance could be improved by the same pathway. 

In recent years, proteomics analysis has helped researchers understand responses to various environmental stresses such as cold [10], drought [11], heat [12,13], flooding [12], and salinity [14]. HT has been found to significantly induce heat-shockprotein expression and inhibit enzyme activities related to redox homeostasis and protein synthesis and degradation in rice, radish, and chickpea [15,16,17,18]. Furthermore, several studies have indicated that LT can downregulate photosynthesis-related proteins and upregulate proteins involved in carbohydrate metabolism, detoxification, ROS scavenging, and cell wall remodeling in wild wheat, *Arabidopsis*, and barley [19,20,21]. Response mechanisms that protect against the potentially harmful effects of HT or LT have been extensively studied in NHCC, but most studies have focused on biochemical responses and specific genes [6,7,22]. Song et al. [23] conducted a comprehensive analysis of responses to LT and HT treatments in NHCC using RNA-sequencing. Among 14,329 differentially expressed genes (DEGs), 33 and 25 genes were enriched by heat and cold stress, respectively. Among the identified DEGs, only 10 were found in response to heat and cold stress. 

In this study, we used tandem mass tag (TMT) to evaluate the molecular changes that occur in the novel NHCC genotype WS-1 in response to LT and HT. TMT is a highly sensitive technique that improves the throughput and dynamic range of protein analysis [24,25]. This study aimed to identify and compare the regulatory mechanisms responsible for tolerance to LT and HT stress in WS-1. 

## 2. Results

### 2.1. Identification of Differentially Expressed Proteins (DEPs) by Quantitative Proteomic Analysis

**Figure 1:**
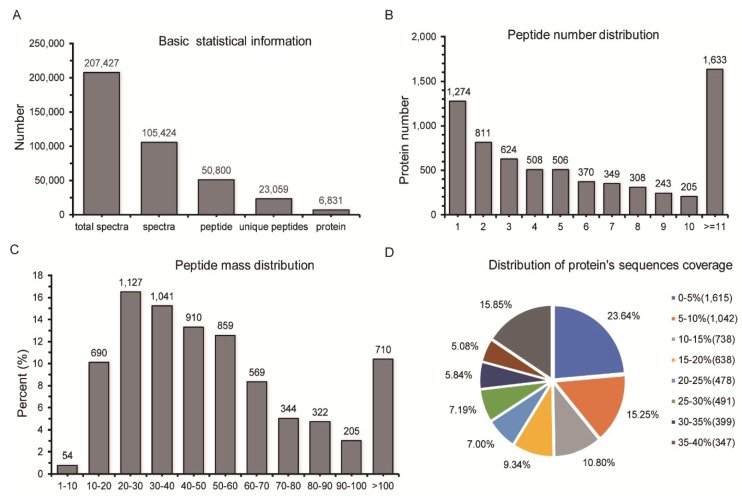
Identification and quantitative evaluation of identified proteins. (**A**) Spectra, peptides, and proteins identified by ProteomeDiscoverer. (**B**) Distribution of peptide numbers as determined by ProteomeDiscoverer. (**C**) Distribution of identified proteins according to molecular mass. (**D**) Distribution of identified protein sequences.

Using liquid chromatography-mass spectrometry (LC-MS/MS), we detected 207,427 total spectra, 105,424 spectra, 50,800 peptides, 23,059 unique peptides, and 6831 proteins (score sequence HT > 0 and unique peptides ≥ 1) (Figure 1A and Supplementary Table S1). The distribution of peptide numbers is shown in Figure 1B, and >81.3% of the proteins had at least two peptides (Figure 1B and Supplementary Table S2). Approximately 99.2% of the proteins had mass >10 kDa, which indicates very good coverage (Figure 1C and Supplementary Table S2). Sequence coverage distribution greater than 10% and 20% were 61.2% and 41.3% respectively, indicating that the data were of high quality (Figure 1D and Supplementary Table S2).

### 2.2. Functional Cataloging of DEPs Common to Both Low-Temperature (LT) and High-Temperature (HT) Groups

**Figure 2:**
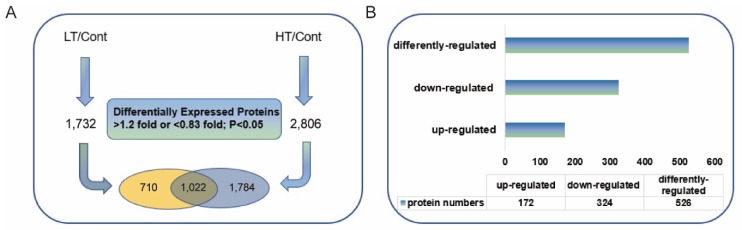
Summary of temperature stress-responsive proteins under low temperature (LT) and high temperature (HT) treatments. (**A**) Number of differentially expressed proteins in wucai leaves under LT and HT treatments compared to control. The value shared by the two ovals indicates the number of commonly regulated proteins, i.e., proteins that were differentially expressed relative to the control under both LT and HT treatments. (**B**) Distribution of commonly regulated proteins (1022 proteins) by LT and HT.

**Figure 3:**
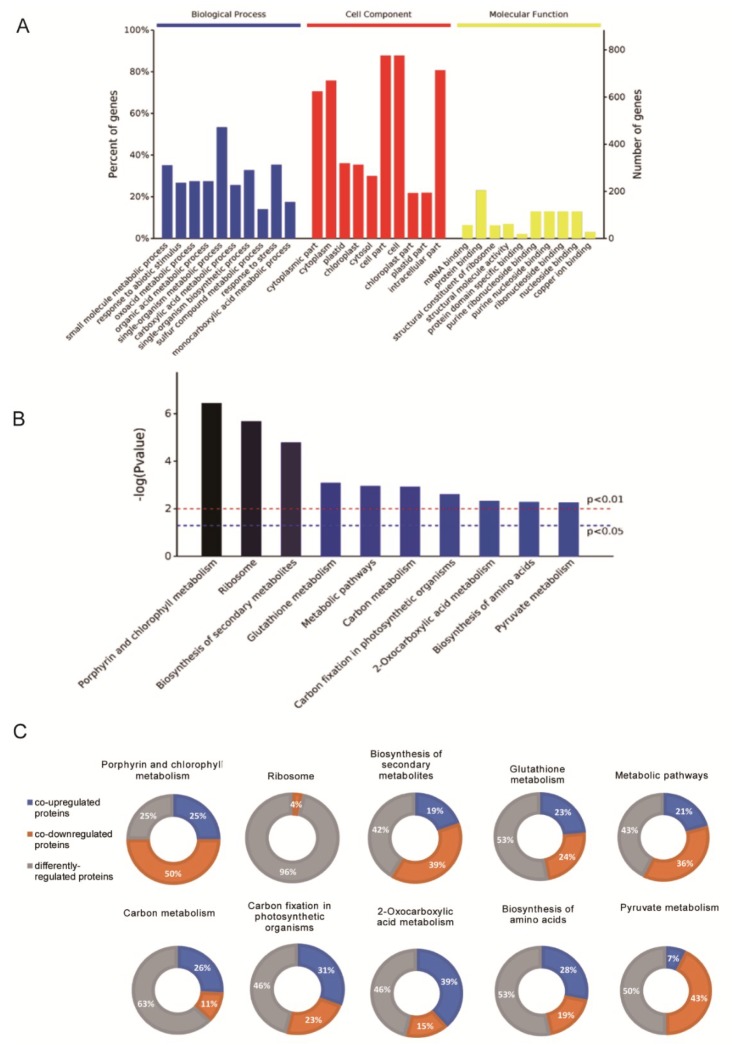
Summary of temperature stress-responsive proteins under LT and HT treatments. (**A**) Histogram presentation of Gene Ontology (GO) classification of 1022 commonly regulated proteins under LT and HT treatments. (**B**) Kyoto Encyclopedia of Genes and Genomes (KEGG) pathways of 1022 commonly regulated proteins. (**C**) Percentage of the 1022 differentially regulated proteins that were co-upregulated (i.e., upregulated under both LT and HT), co-downregulated (i.e., downregulated under both LT and HT), and differently regulated (upregulated in response to one temperature treatment but downregulated in response to the other) in each KEGG pathway.

We regarded proteins in the LT or HT treatment with abundance >1.2-fold and *p* < 0.05 relative to the control treatment as upregulated, and those with abundance <0.83-fold and *p* < 0.05 as downregulated. A total of 1,732 differentially expressed proteins (DEPs) were identified in the LT treatment, and 2806 DEPs were identified in the HT treatment (Figure 2A). These upregulated and downregulated DEPs were assigned to three groups: (1) 710 DEPs were specific to the LT group, (2) 1022 DEPs were common to both the LT and HT groups, including 172 common upregulated proteins, 324 common downregulated proteins, and 526 differently regulated proteins (Figure 2B), and (3) 1784 DEPs were specific to the HT group. To determine the mechanism underlying the temperature stress tolerance of WS-1, we focused on the functions of the 1022 DEPs that were regulated under both LT and HT conditions.

The functions of these “commonly regulated” proteins were assigned to several groups based on their Gene Ontology (GO) annotations (Figure 3A, Supplementary Tables S1 and S2), including biological process, cell component, and molecular function. As expected, several DEPs were assigned to multiple groups. In the LT/Cont treatment, single-organism metabolic process, small-molecule metabolic process, and single-organism biosynthetic process were the main GO enrichment pathways (Supplementary Table S1). Single-organism cellular process, response to stimulus, and single-organism metabolic process were the predominant pathways in the HT/Cont treatment (Supplementary Table S2). Biological process analysis of 1022 DEPs, commonly regulated proteins in LT/Cont and HT/Cont treatments, suggested that the predominant pathways were small-molecule metabolic process and response to abiotic stimulus. Most of the commonly regulated DEPs participate in various molecular metabolic processes (small-molecule, oxoacid, organic acid, single-organism, carboxylic acid, sulfur compound, and monocarboxylic acid), while the remainder are involved in response to stimulus. As shown in Figure 3A, and Supplementary Tables S1 and S2, most of the commonly regulated DEPs were predicted to localize in the cell part, cell, intracellular part, and cytoplasm, the same as in the LT/Cont and HT/Cont treatments, indicating that DEPs mainly functioned on these cell components. The main molecular functions of commonly regulated DEPs were RNA binding and protein binding in the HT/Cont treatment, whereas they were catalytic activity and RNA binding in LT/Cont (Figure 3A, Supplementary Tables S1 and S2).

To identify the metabolic pathways relevant to temperature stress tolerance, 1022 DEPs were further analyzed according to the Kyoto Encyclopedia of Genes and Genomes (KEGG) database (Figure 3B). Among the 1022 commonly regulated DEPs, 172 DEPs were upregulated in both LT and HT treatments, 324 were downregulated in both treatments, and 526 were differently regulated (Figure 2B). Most DEPs were enriched in porphyrin and chlorophyll metabolism, ribosome (4% of the downregulated DEPs), biosynthesis of secondary metabolites, glutathione metabolism, metabolic pathways, carbon metabolism, carbon fixation in photosynthetic organisms, 2-oxocarboxylic acid metabolism, biosynthesis of amino acids, and pyruvate metabolism (Figure 3C). Upregulated DEPs were enriched in 2-oxocarboxylic acid, carbon fixation in photosynthetic organisms, biosynthesis of amino acids, and carbohydrate metabolism.

### 2.3. Functional Cataloging of DEPs That Were Upregulated under both LT and HT

**Figure 4:**
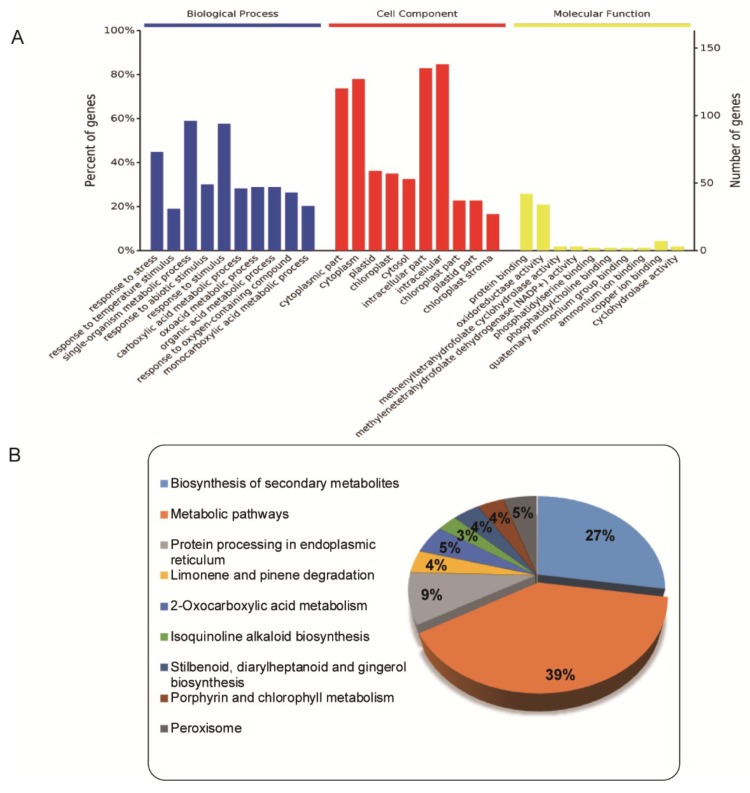
Summary of 172 proteins that were upregulated in LT and HT treatments. (**A**) Histogram of GO classification of upregulated proteins under both treatments. (**B**) Percentage of the 172 upregulated proteins in each KEGG pathway.

A total of 172 DEPs that were upregulated under both LT and HT were assessed using GO annotation and KEGG pathway analysis. GO analysis was used to assign these DEPs to three categories: biological processes, cell components, and molecular functions (Figure 4A). For biological processes, the analysis suggested that the 172 DEPs, which were predicted to be located in the cytoplasm and in chloroplasts and plastids, were involved in response to abiotic stimulus and stress, and in the metabolic process of carboxylic acid, oxoacid, organic acid, and monocarboxylic acid. In molecular functions, the most prevalent categories were protein binding and oxidoreductase activity. The KEGG pathway analysis indicated that most of the proteins were enriched in the secondary metabolite biosynthesis and metabolic pathway (Figure 4B). A more detailed ontological analysis of the response to temperature stress was then obtained for the 172 DEPs that were upregulated in both LT and HT treatments. According to GO annotation, these upregulated DEPs were divided into several metabolic groups: redox homeostasis, photosynthesis, carbohydrate metabolism, heat-shock proteins, signal transduction, and metabolic process (Supplementary Table S1). Because not all proteins have been identified in wucai, some unidentified proteins were mapped to the *Arabidopsis* genome.

#### 2.3.1. Proteins Involved in Redox Homeostasis

Of the 172 DEPs that were upregulated in both LT and HT treatments, those that were characterized were disulfide-isomerases, amine oxidase, isocitrate dehydrogenase (NADP) (ICDH) (A0A078FVI0), delta-1-pyrroline-5-carboxylate synthase (P5CS) (M4DE13), and ubiquinol oxidase (AOX) (J7GUS8) (Supplementary Table S3). The uncharacterized proteins—ferredoxin-dependent glutamate synthase (Fd-GOGAT) (A0A0D3EHZ6), glutathione hydrolase (A0A0D3BW42), betaine aldehyde dehydrogenase (ALDH) (A0A0D3CWL2), and ubiquinol oxidase (AOX) (J7GUS8)—were mapped to respective genes: *GLU1*, *GGT1*, *ALDH10A8*, and *AOX1A*. Among the proteins in the redox homeostasis category, upregulation was highest for GLU1: 3.46-fold under LT and 2.51-fold under HT.

#### 2.3.2. Proteins Involved in Photosynthesis

Several crucial DEPs were categorized in the photosynthesis pathway (Supplementary Table S3). Two proteins related to the oxygen-evolving complex, PSBO1 (M4F7V3) and PSBP1 (A0A078FRX3), were mapped, and among the 172 commonly upregulated proteins, the fold increase was highest for *PsbO1*: upregulation was 18.87-fold under LT and 21.88-fold under HT. In addition, NADPH-protochlorophyllide oxidoreductase (PORC) (M4EX79) was upregulated and is involved in pigment metabolism.

#### 2.3.3. Proteins Involved in Carbohydrate Metabolism

Several crucial enzymes involved in starch and sucrose metabolism, including beta-amylase (BAM) (M4DEV3) and sucrose synthase (SUS) (M4CQT7), were also enhanced under LT and HT (Supplementary Table S3).

#### 2.3.4. Heat-Shock Proteins and Chaperones

Putative small heat-shock proteins and chaperones were upregulated in response to LT and HT treatments: M4DG78, M4CEA8, M4CQW7, A0A0D3EDG5, M4F2A3, A0A0D3CBS0, and M4DH02 (Supplementary Table S3). These proteins were mapped to genes *Hsp17.7*, *Hsp18.1*, *ATJ2*, *ATJ3*, *DJA7*, *ERD10*, and *ERD14* respectively, in the *Arabidopsis* genome.

#### 2.3.5. Proteins Relevant to Stimulus and Signaling Transduction

The signal transduction pathway from the initial perception of LT or HT to the final adaptive stress-responsive change in protein expression is very complex (Supplementary Table S3). Several characterized proteins thiamine thiazole synthase (THI) (A0A078I0E1) and UDP-glycosyltransferases (UGTs) (A0A0D3B6L4) and several uncharacterized proteins mapped to abscisic acid (ABA) and salicylic acid (SA) metabolism BAX inhibitor 1 (BI-1) (A0A0D3AUW9), BURP domain protein (RD22) (A0A078JCL4), and glycine-rich protein (GRP3) (A0A078INC4) were upregulated in response to both LT and HT.

#### 2.3.6. Metabolic-Related Proteins

CytochromeP450s (CYPs) participate in the oxidation of lipophilic substrates as heme-thiolate proteins. Proteins mapped to the CYP family—A0A078IGH2, M4C991, M4E8T8, A0A078HAQ8, M4DYH4, A0A078HUG7, and A0A078GN44—accounted for almost half of this category. Their corresponding genes were *CYP38*, *CYP706A6*, *CYP71B3*, *CYP71B35*, *CYP72A15*, *CYP71B4*, and *CYP71B28*, respectively (Supplementary Table S3). The abundance of these proteins significantly increased in response to both LT and HT treatments.

#### 2.3.7. Protein–Protein Interaction (PPI) Analysis

**Figure 5:**
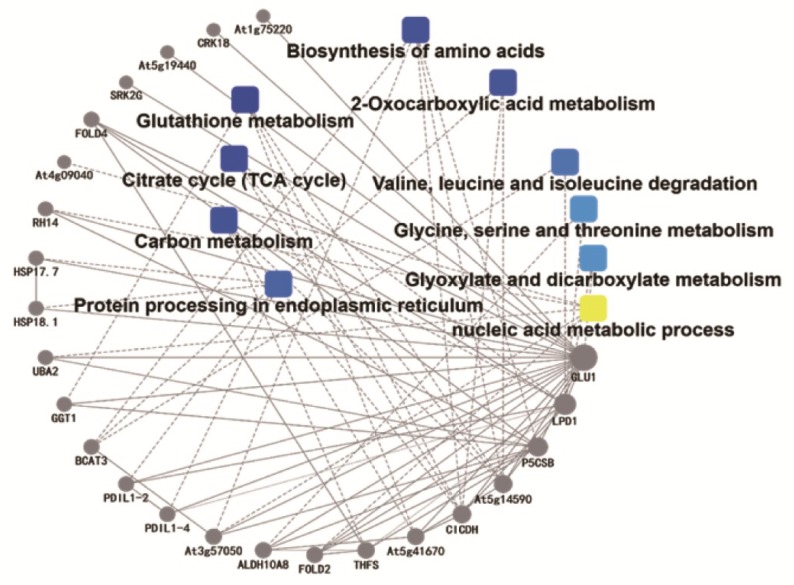
Protein–protein interaction (PPI) network analysis by the Search Tool for the Retrieval of Interacting Genes/Proteins (STRING) PPI network. 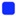, GO/KEGG term; 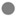, protein. Symbol color corresponds to degree of interaction.

The present study used the Search Tool for the Retrieval of Interacting Genes/Proteins (STRING), a biological database and web resource of known and predicted protein–protein interactions (PPI). STRING PPI network analysis revealed the functional partnership and interaction networks. It was found that glutathione metabolism occupied a dominant position among all metabolic pathways. In addition, GLU1 had direct or indirect correlations with other metabolic processes, of which the major ones were SRK2G (P43292), FOLD4 (A0A0D3DZN5), RH14 (Q8H136), HSP17.7 (M4DG78), HSP18.1 (M4CEA8), GGT1 (A0A0D3BW42), BCAT3 (Q9M401), PDIL1-2 (Q9SRG3), ALDH10A8 (A0A0D3CWL2), ICDH (A0A078FVI0), and P5CSB (M4DE13) (Figure 5).

### 2.4. Expression Profiles of Genes Involved in DEPs That Were Upregulated under Both LT and HT

**Figure 6:**
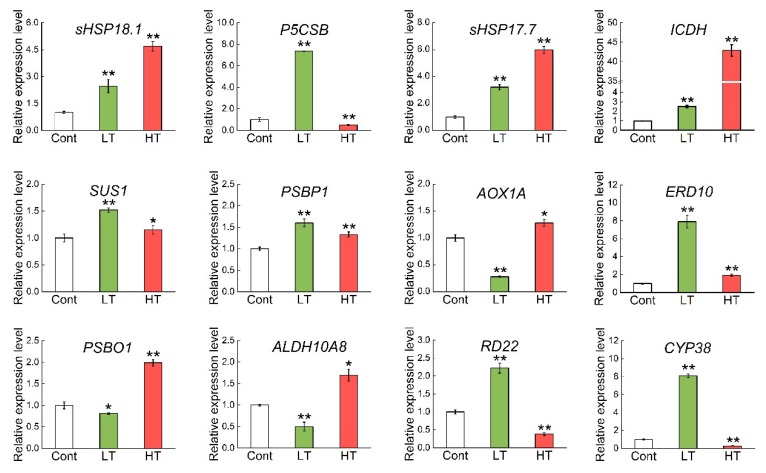
Gene expression related to upregulated differentially expressed proteins (DEPs) in leaves of plants subjected to LT and HT. *Actin* was used as the internal control to calculate the relative expression level. Data shown here are mean ± standard deviation (SD) of 3 biological replicates. ** Significant difference *at p* < 0.01.

To evaluate the correlation between mRNA and protein expression levels, we selected 12 genes related to DEPs that were upregulated under both LT and HT for real-time polymerase chain reaction (RT-PCR) analysis. The results show that the expression of most of these genes was greater in the LT or HT group than in the control group. The expression of the following genes was higher in the LT group: *RD22*, *SUS1*, *P5CSB*, and *CYP38* (Figure 6). Compared to the control, expression levels of *sHSP17.7*, *sHSP18.1*, *ICDH*, *PSBP1*, and *ERD10* were significantly increased in response to both LT and HT treatments. These results indicate that the protein expression patterns were not always consistent with their transcriptional expression patterns, perhaps because stress-related genes dominated the response to LT and HT.

### 2.5. Overexpression of BccrGLU1 in Arabidopsis Leads to Temperature Stress Tolerance

**Figure 7:**
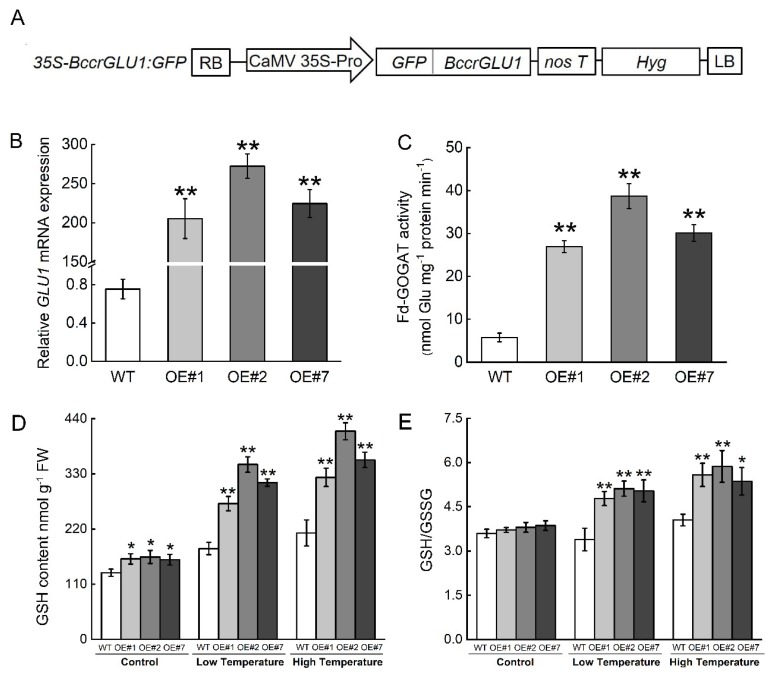
Schematic diagram of *35S-BccrGLU1:GFP* fusion protein construct and physiological changes associated with temperature stress response in wild-type (WT) and overexpressed (OE) lines of *Arabidopsis* plants. (**A**) Schematic diagram of *35S-ccrGLU1:GFP* fusion protein construct. (**B**) Relative expression of *BccrGLU1* in T3 transgenic plants. WT: Col-0; OE#1, OE#2, OE#7. T3 plants with *BccrGLU1* on the *AtCol-0* background. (**C**) Fd-GOGAT activity in WT and OE lines. (**D**) Glutathione (GSH) content in leaves. (E) GSH/oxidized glutathione (GSSG) ratio in leaves. Values represent mean ± SE (*n* = 3). ** and * indicate significant differences from wild-type plants at the level of *p* < 0.01 and *p* < 0.05 respectively, using Student’s t-test.

To investigate the effect of redox homeostasis on tolerance to temperature stress in plants, we generated transgenic *Arabidopsis* plants overexpressing *BccrGLU1* under the control of the CaMV 35S promoter (Figure 7A). Quantitative RT-PCR results show that the transcript level of *BccrGLU1* was highly induced in selected transgenic plants, confirming that the plants had been successfully transformed with *35S-BccrGLU1:GFP* (Figure 7B). No visible phenotype differences between wild-type (WT) and overexpressed (OE) transgenic lines were detected under normal growth conditions, indicating that overexpression of *BccrGLU1* did not cause phenotypic defects in transgenic *Arabidopsis* seedlings. In OE lines, ferredoxin (Fd)-GOGAT activity was significantly higher than in WT under normal conditions (Figure 7C).

To evaluate the role of *BccrGLU1*, physiological assays were performed with three OE lines. Glutathione (GSH) levels were higher in OE lines than in WT under normal conditions. Under low and high temperature, GSH levels in three overexpression plants (OE#1, OE#2, and OE#7) were significantly higher than in wild-type plants (Figure 7D). This increased degree in OE lines under temperature stress was greater than under normal conditions. The GSH/oxidized glutathione (GSSG) ratio in OE lines was not different from WT under normal conditions, while the ratio in OE lines was significantly higher than in WT plants under temperature stress (Figure 7E).

**Figure 8:**
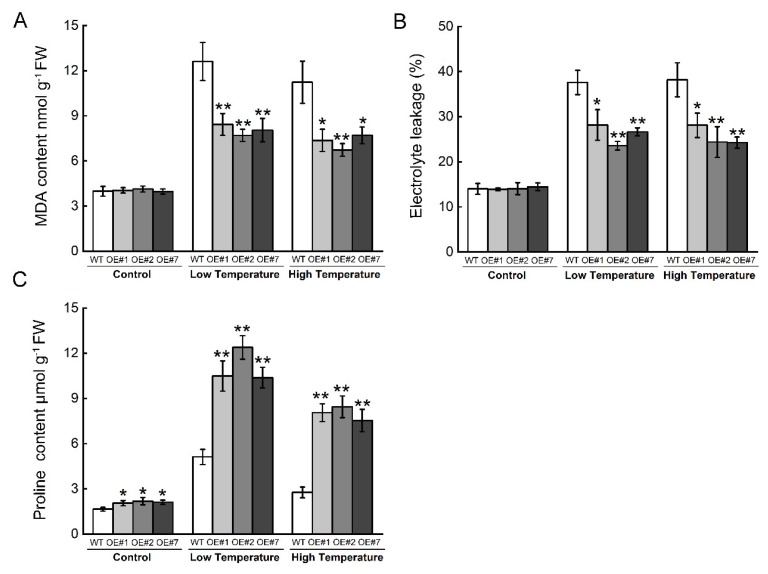
Oxidative damage and proline content in WT and OE lines of *Arabidopsis* plants. (**A**) Malondialdehyde (MDA) content in *Arabidopsis* seedlings. (**B**) Electrolyte leakage in *Arabidopsis* seedlings. (**C**) Proline content in *Arabidopsis* seedlings. Values represent mean ± SE (*n* = 3). ** and * indicate significant differences in wild-type plants at the level of *p* < 0.01 and *p* < 0.05 respectively, using Student’s t-test.

In addition, we determined oxidative damage parameters under temperature stresses to evaluate the redox homeostasis in OE lines. Under normal conditions, MDA content and electrolyte leakage in OE lines were not different than those in WT. When exposed to LT and HT, OE lines had lower MDA content and electrolyte leakage than WT (Figure 8A,B). Proline content in three OE lines was slightly higher than in WT under normal conditions, and proline content of OE lines had greater increases than WT responding to LT and HT (Figure 8C).

**Figure 9:**
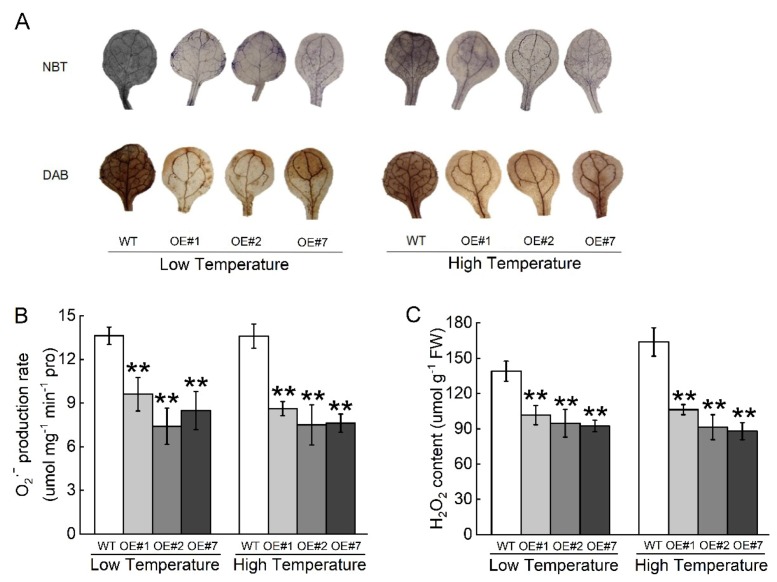
Oxidative damage of WT and OE lines of *Arabidopsis* plants. (**A**) Histochemical staining assay for superoxide anion (O_2_^•−^) by nitro blue tetrazolium (NBT) and hydrogen peroxide (H_2_O_2_) in the upper row and by 3,3-diaminobenzidine (DAB) staining in the lower row. (**B**) Quantification of O_2_^•−^ content. (**C**) Quantification of H_2_O_2_ content. Values represent mean ± SE (*n* = 3). ** indicate significant differences from wild-type plants at the level of *p* < 0.01, using Student’s t-test.

The staining patterns indicated lower ROS levels in OE lines compared to WT under stress (Figure 9A). The hydrogen peroxide (H_2_O_2_) content and O_2_^.−^ generation rates were also decreased in OE lines compared with wild-type, which was consistent with the staining pattern (Figure 9B,C). These results indicate that *BccrGLU1* was associated with reactive oxygen species scavenging to counteract oxidative stress.

## 3. Discussion

The mechanisms that enable plants to tolerate LT and HT are morphological, physiological, and molecular in nature and often vary among genotypes. The novel genotype WS-1, which is tolerant to both LT and HT, could facilitate the breeding of temperature tolerance in wucai [8,9]. Because protein metabolic processes are very susceptible to environmental temperature, proteomic research could greatly increase our understanding of the protein alteration response to temperature stress. In the present study, we found that redox homeostasis, photosynthesis, carbohydrate metabolism, heat-shock protein, and signal transduction pathways were associated with temperature stress tolerance in WS-1.

In the current research, 172 “commonly upregulated” DEPs (i.e., DEPs that are upregulated in response to both LT and HT) in WS-1 were identified to respond to LT and HT (Supplementary Table S1). There were significantly more DEPs than in previous transcriptome analysis of another NHCC variety according to Reference [23], which found seven upregulated and three downregulated in response to heat and cold stress, and most of the DEGs were involved in stress-related protein, such as *KIN2* and *LEA14*. In our study, more than 20 DEPs were assigned to the redox homeostasis pathway. The putative Fd-GOGAT protein, mapped to *GLU1*, was upregulated under both LT and HT. In a previous report, a *GLU1*-deficient mutant of *Arabidopsis* displayed a lethal phenotype, indicating that *GLU1* is a major gene that encodes Fd-GOGAT [26]. As indicated in another report [27], Fd-GOGAT might contribute significantly to photosynthesis, and its upregulation in response to LT and HT in the current study may enhance the production of glutathione. The abundance of putative gamma-glutamyl transferase (GGT) was enhanced in the current study and was mapped to *GGT1*. GGT was also found to be involved in glutathione metabolism, which can synthesize and degrade the glutathione in the gamma-glutamyl cycle [28]. In addition, ICDH, which plays a crucial role in the oxidative pentose phosphate pathway, was upregulated in response to LT and HT in our study. Redox-proteomics analysis also verified that ICDH is a key enzyme in the tricarboxylic acid cycle as a potential redox-regulated protein in *Arabidopsis* [29]. The upregulation of these DEPs in response to LT and HT indicates that glutathione metabolism has a dominant role in the redox balance of WS-1 plants exposed to low- or high-temperature stress.

Glycine betaine (GB) and proline, commonly referred to as osmoprotectants, protect plants from abiotic stress by contributing to cellular osmotic adjustment, detoxification of ROS, protection of membrane integrity, and stabilization of enzymes/proteins [30,31]. In the present study, two upregulated genes in WS-1, *ALDH10A8* and *P5CSB*, were identified to code for BADH and P5CS under LT and HT. Consistent with our results, *ALDH10A8* mutant was more sensitive to dehydration and salt stress than wild-type plants, indicating that the *ALDH10A8* gene can respond to abiotic stress in *Arabidopsis* [32]. Under cold stress, the *BADH* transgenic lines with higher GB content maintained better membrane integrity and had lower ROS production, which was attributed to enhanced cold stress [33]. In yet another study, *Arabidopsis* with an antisense proline dehydrogenase cDNA exhibited increased accumulation of proline and a constitutive tolerance to freezing temperatures (as low as −7 °C) [34]. Proline has been verified to function in multiple ways as a molecular chaperone, which can protect protein integrity and increase many enzyme activities [31]. *P5CS2* in *Arabidopsis* can be activated by bacteria, salicylic acid, and reactive oxygen species signals [35]. In the present study, we that found P5CS protein, encoded by *P5CSB*, which is homologous to *P5CS2*, was enhanced in response to temperature stress. Overexpressing *BccrGLU1 Arabidopsis* plants with a higher proline content in our study also exhibited stronger tolerance to temperature stress compared with WT (Figure 8C). Therefore, an increased abundance of BADH and P5CSB may modify hormone signal transduction in order to achieve redox balance in response to temperature stress. In agreement with this, an enhanced abundance of putative AOX in response to LT and HT mapped to *AOX1a*, also contributed to the intercellular redox homeostasis [36].

Among the upregulated proteins related to photosynthesis under both LT and HT, the abundance of the putative oxygen-evolving complex proteins PsbO and PsbP showed a large increase relative to the control. A *PsbO*-deficient mutant of *Arabidopsis* had retarded development, pale leaves, and increased sensitivity to light stress [37]. The PsbO protein, however, is downregulated under LT stress in *Spirulina*, which causes a decreased electron transfer rate and photosynthetic capacity [38]. This supports that PsbO plays a dominant role in protecting and stabilizing the catalytic center as an important protein for Mn_4_Ca cluster stabilization [39]. An additional protein mapped to *PsbP1*, which is involved in electron transfer within PSII [40], was also increased in response to HT and LT stress in the current study. Researchers have speculated that the nitratable tyrosine residues of PsbO1 and PsbP1 are redox-active and sensitive to ROS [41]. Thus, the relatively stable redox state might facilitate the expression of PsbO and PsbP.

Enhanced expression of SUS and BAM proteins in the wucai genotype WS-1 was observed in the current study, confirming that LT and HT enhance the expression of these proteins. Expression of the gene encoding SUS is induced by cold in sugar beet [42], and an increased accumulation of soluble sugars has been documented in chickpea subjected to cold treatment [17]. Increased BAM abundance in WS-1 was favorable to catalyze the breakdown of starch to generate maltose and participate in the metabolism of sugar [43]. Our findings are consistent with previous reports that attributed the enhanced thermal tolerance of *Agrostis* grass species to the upregulation of enzymes involved in sucrose metabolism [44].

According to GO annotation, several uncharacterized DEPs are mapped to the following heat-shock proteins, heat-shock-related proteins, and chaperones: HSP17.7, sHSP18.1, ERD10, ERD14, DJA7, ATJ2, and ATJ3. These proteins were upregulated in both LT and HT treatment. Researchers have also reported that sHSPs can refold proteins that were denatured by freezing so as to prevent the aggregation of denatured proteins under cold stress [45,46]. Supporting this, transgenic carrot cell lines with *sHsp17.7* have an enhanced survival rate at high temperatures [47]. In peas, heat-denaturing proteins that initially interact with *sHSP18*.1 have remarkably improved subsequent refolding by *HSP70* [48]. Although the function of molecular chaperones *sHSP18.1* and *sHSP17.7* is not clear, the results from our study strongly support the notion that both sHSP18.1 and sHSP17.7 enhance the thermotolerance of WS-1. The identification of several unique small HSPs in our study suggests that these could now be used as biomarkers for evaluation of thermal-stress tolerance in wucai [15,49].

Several uncharacterized proteins that were mapped to *BI-1*, *RD22*, *GPR3*, and *UGTs* were related to plant hormone signal transduction under both LT and HT treatment. *BI-1* expression in *Arabidopsis thaliana* is induced by exogenous SA, and its overexpression increases the viability of rice cells after application of SA [50,51]. Wang et al. [52] demonstrated that overexpression of the soybean protein *GmRD22* in rice could be involved in the ABA regulation pathway [53]. *UGT73B5* belongs to the early SA-induced genes, whose pathogen-induced expression is co-regulated with genes related to cellular redox homeostasis [54]. The involvement of THI, which is required for plant development, has been implicated in ABA signaling in *Arabidopsis* guard cells [55] and salt stress [56]. These studies suggest that plant hormone signaling contributes to temperature-stress tolerance in WS-1.

Based on the above analysis and PPI network annotation, we considered that GLU1 has strong interactions with the proteins that contribute to several protein metabolic processes when encountering temperature stresses. Then we generated *35S:BccrGLU1* overexpression transgenic lines in *Arabidopsis*. In OE lines and WT, the glutathione redox status was assayed by measuring GSH and GSSG levels in two-week-old *Arabidopsis* seedlings. GSH is not only a key metabolite in the removal of ROS but also can control the expression of redox-sensitive proteins, which could alter the intercellular redox state and induce tolerance of ROS-dependent signaling pathways [57]. Higher GSH has been shown to positively modulate tolerance to HT stress in mung beans [58] and to LT stress in maize [59]. In the present study, GSH content in three OE lines was strongly induced, whereas GSSG had slight increases compared with WT under LT and HT. The higher ratio of GSH/GSSG in OE lines was beneficial to antioxidative protection, which could in turn improve thermotolerance [60]. In the *Arabidopsis cat2* mutant, in which the GSH/GSSG ratio is altered, glutathione helps regulate the oxidant-dependent salicylic acid and jasmonic acid signaling pathways [61,62]. In addition, compared to WT, OE lines exhibited lower MDA content and electronic leakage, supported by the lower H_2_O_2_ content and O_2_^·−^ generation rate in response to temperature stresses. Higher proline content in OE lines reduced oxidative injury by lowering free radical generation as important osmolytes [63]. This provides evidence that *BccrGLU1* contributes to balance redox homeostasis in WS-1 to confer temperature stress response. Moreover, it is consistent with our proteomic data, where most of the identified DEGs were associated with or modulated by redox homeostasis.

## 4. Materials and Methods

### 4.1. Plant Materials and Growth Conditions

WS-1, which has stable genetic traits, was obtained by multigenerational self-inbreeding via manual pollination at Anhui Agricultural University. WS-1 seeds were sown in a peat/vermiculite (2:1) medium, and the seedlings were kept in a growth chamber at 25 ± 1 °C (day) and 18 ± 1 °C (night), with relative humidity of 70% and a 14/10 h light/dark photoperiod with 300 μmol·m^−2^·s^−1^ photosynthetically active radiation. After 20 days, the seedlings had developed 4−5 true leaves and were randomly divided into 3 groups (50 seedlings per group). Each group was subjected to 1 of 3 treatments: control (25/18 °C day/night), LT (3/8 °C), or HT (40/30 °C). The experiment, which used 3 growth chambers, had a completely randomized design and was repeated 3 times, such that each treatment was assigned to the same growth chamber only once. Thus, each repetition of the experiment was considered a block, for a total of 3 blocks.

After the seedlings were exposed to the treatments for 3 days, the leaves from each of 6 randomly selected plants per treatment group were collected for proteomics analysis and assessment of physiological properties.

### 4.2. Protein Extraction

For extraction of proteins, the leaf samples were crushed in 500 μL of extraction buffer before Tris-phenol buffer was added, and the preparation was mixed at 4 °C for 30 min. After the mixture was centrifuged at 7100× *g* and 4 °C for 10 min, the supernatant was collected. Five volumes of 0.1 M cold ammonium acetate–methanol buffer was added to the supernatant, which was precipitated at –20 °C for 12 h. The precipitated sample was centrifuged at 12,000× *g* and 4 °C for 10 min, and the supernatant was removed to obtain the precipitate. The precipitate was washed with cold methanol, gently mixed, and centrifuged again to collect additional precipitate. The methanol was then replaced with acetone to remove the methanol contaminants, and the steps used to obtain precipitate were repeated. The precipitate was dried at room temperature, mixed with lysis buffer, and dissolved for 3 h. Finally, the sample was centrifuged at 12,000× *g* and 4 °C for 5 min, and the supernatant was retained. The supernatant was subjected to a second centrifugation to completely remove the precipitate. Protein concentration was determined using the Bradford assay [64], and aliquots were stored at −80 °C.

### 4.3. Protein Reduction, Alkylation, Digestion, and TMT Labeling

The filter-aided sample preparation (FASP) method was used for protein reduction [65]. The steps of trypsin digestion were carried out as described by Cen et al. with partial modification [66]. First, 100 μg of protein extract was added to 120 μL of reduction buffer (10 mM DTT, 8 M urea, 100 mM triethylammonium bicarbonate (TEAB), pH 8.0). The solution was incubated at 60 °C for 1 h and iodoacetamide (IAA) was added to the solution with the final concentration of 50 mM in the dark for 40 min at room temperature. The protein was then digested and centrifuged at 10,000× *g* for 20 min at 4 °C and the flow-through was discarded from the collection tube. Then, 100 μL of 100 mM TEAB was added to the solutions and centrifuged at 10,000× *g* for 20 min, and this step was repeated twice. The filter units were transferred into new collection tubes and 100 μL of 100 mM TEAB was added, followed by 2 μL of sequencing-grade trypsin (1 μg·μL^−1^) in each tube. Then the solutions were incubated for digestion at 37 °C for 12 h. Finally, the collections of digested peptides were centrifuged at 10,000× *g* for 20 min. After 50 μL of TEAB (100 mM) was added, the preparation was centrifuged again, and the solution was lyophilized and stored at −80 °C.

For TMT labeling, 100 μL of TEAB (50 mM) was added to the sample, and 40 μL of the sample was used for labeling. A 60 mL volume of TEAB was added to the 40 μL sample and mixed well, before 40 μL of anhydrous acetonitrile was added to the TMT reagent vial. The solution was thoroughly dissolved for 5 min and centrifuged at 10,000× *g* and 4 °C for 30 min. Next, 41 μL of TMT labeling reagent was added to 100 μL of sample and mixed well. The tubes were incubated at room temperature for 1 h. Finally, 8 µL of 5% hydroxylamine was added to each sample and incubated for 15 min to terminate the reaction. The solution was incubated for 1 h at room temperature. Finally, the reaction was terminated by adding 8 μL of 5% hydroxylamine to the sample. The labeling peptide solutions were lyophilized and stored at −80 °C. The samples were labeled with TMT tags as 126 (control), 127N (control), 127C (control), 128N (HT), 128C (HT), 129N (HT), 129C (LT), 130N (LT), and 130C (LT).

### 4.4. Reversed-phase liquid chromatography (RPLC) Analysis

Reversed-phase (RP) separation was performed on an Agilent 1100 HPLC System (Agilent Technologies Inc., CA, USA) using an Agilent Zorbax Extend RP column (5 μm, 150 mm × 2.1 mm). The RP separation was conducted with mobile phases A (2% acetonitrile in high-performance liquid chromatography (HPLC) water) and B (98% acetonitrile in HPLC water) as follows: 0−8 min, 98% A; 8.00−8.01 min, 98−95% A; 8.01−38 min, 95−75% A; 38−50 min, 75−60% A; 50−50.01 min, 60−10% A; 50.01−60 min, 10% A; 60−60.01 min, 10−98% A; and 60.01−65 min, 98% A. Tryptic peptides were separated at a fluent flow rate of 300 μL·min^−1^ and were monitored at 210 and 280 nm. Dried samples were harvested for 8−50 min, and the elution buffer was collected once per minute and numbered from 1 to 10 with pipeline. The separated peptides were lyophilized for MS detection.

### 4.5. Liquid Chromatography-Mass Spectrometry Analyses

LC-MS/MS was carried on an Agilent 1100 HPLC purifier system (Agilent Technologies Inc., CA, USA) according to the method of Cao et al. with slight modification [24]. The samples were loaded on a trap column (100 µm × 20 mm; RP-C18, Thermo Scientific Inc., MA, USA) with an autosampler and separated with an analysis column (75 µm × 150 mm; RP-C18, Thermo Scientific Inc.) at 300 nL·min^−1^. The mobile phase consisted of buffer A (0.1% formic acid in water) and buffer B (0.1% formic acid in acetonitrile). The linear washing gradient was set as follows: 0–7 min, 4% buffer B; 8–51 min, 4–25% B; 52–60 min, 25–40% B; 61–70 min, 40–85% B; 71–74 min, 85% B. Full MS scans were acquired in the mass range of 375–1800 *m*/*z* with a mass resolution of 120,000 (at *m*/*z* 200), the automatic gain control (AGC) target value was set at 4 × 10^5^, and the maximum injection time was 50 ms. The dynamic exclusion was set to 60.0 s and run under positive mode. The 10 most intense peaks in MS were fragmented with higher-energy collisional dissociation with collision energy of 38 eV. MS/MS spectra were obtained with a resolution of 50,000 (at *m*/*z* 200) and max injection time of 100 ms.

### 4.6. Protein Identification and Quantification

Proteome Discoverer (v.2.2, Thermo Fisher Scientific, Bremen, Germany) was used to thoroughly search all of the fusion raw data against the sample protein database. Database searches were carried out with trypsin digestion in *Brassica fasta*. Alkylation on cysteine was considered to be a fixed modification in the database search. The analysis results were filtered as follows: significance threshold *p* < 0.05 (with 95% confidence) and ion score or expected cutoff <0.05 (with 95% confidence).

The main parameters were set as per the method of Xie et al. [67]. TMT 10-plex was selected for the protein quantification method. A global false discovery rate was set at 0.01, and protein groups required at least 2 peptides to be considered for quantification. For protein quantification, the protein ratios were calculated as the median of only the unique peptides of the protein. All peptide ratios were normalized by the median protein ratio. Cutoff values of >1.20- and <0.83-fold were used to identify upregulated and downregulated proteins at *p* < 0.05.

### 4.7. Bioinformatics Analysis of Proteins

Bioinformatics analysis of proteins was performed as previously reported [68]. The proteins were described by searching against the UniProt database (28 January 2018; 172,630 sequences). GO (http://www.geneontology.org/) function entries for all aligned protein sequences were extracted using the mapping function of BLAST2GO (version 3.0). The DEPs were further analyzed using KEGG (http://www.genome.jp/kegg/) to determine the active biological pathways [69]. The chi-square test with a defining cutoff of 0.01 was performed to evaluate the functional category protein enrichment. A false discovery rate significance threshold of 0.05 was used as false-positive control. The STRING database (version 9.1; http://string-db.org/) was used to analyze the protein–protein interaction (PPI) networks among these DEPs.

### 4.8. Transcriptional Expression Analysis by Quantitative RT-PCR

Based on the functional category and the fold change in expression in the LT and HT groups relative to the control group, 15 genes were chosen for qRT-PCR analysis. Primer software version 5.0 (Premier Biosoft International, CA, USA) was used to design gene-specific primers, and primer sequences are indicated in Table 1. qRT-PCR analysis was performed using AceQ qPCR SYBR GREEN Master Mix (Vazyme Biotechnology Co., Nanjing, China), and relative expression levels were calculated using the 2^−^^△△*C*t^ method [70]. The *BnaActin* gene was used as the control. Each experiment was replicated 3 times.

### 4.9. Tolerance Assay in Transgenic Arabidopsis under Temperature Stress

The putative Fd-GOGAT protein, which was upregulated under HT and LT stress, was selected to confirm gene function. Fd-GOGAT, one type of GOGAT, is specifically distributed in photosynthetic organisms and has a major role in photosynthetic tissues [71]. There are 2 genes in *Arabidopsis* that encode Fd-GOGAT, GLU1 and GLU2. It has been proven that GLU1 is highly expressed, primarily in leaves [72]. To generate *35S:GFP-BccrGLU1/*Col-0 lines, the Co-Ding Sequence (CDS) encoding the full-length sequence of *BccrGLU1* were amplified from cDNA using gene-specific primers (LP, 5′-gggacaagtttgtacaaaaaagcaggcttcATGATTATTAATTTAATATACATATATGTAGCTGCGC-3′; RP, 5′-ggggaccactttgtacaagaaagctgggtcGTCGAAATTGACACCATACTTGGG-3′). Then, the fragments were introduced into the pDONR207 entry vector with BP clonase (Invitrogen, Carlsbad, CA, USA). LR clonase (Invitrogen) reaction was performed to transfer the inserted fragments to destination vector pMDC43 for GFP fusion. *35S*-*BccrGLU1:GFP* was transformed into WT *Arabidopsis* plants Col-0 by Agrobacterium (strain GV3101)-mediated transformation via floral dip [73]. OE were screened on 1/2 MS agar plates containing 50 µg/mL hygromycin B (Invitrogen). Quantitative RT-PCR was carried out to determine the expression level of transgene using gene-specific primers (LP, 5′-TCCAAGAGAACCAGACGCAGA-3′ and RP, 5′-TTTGCTATAAACCCGACACCAC-3′).

To determine temperature stress tolerance of the transgenic *Arabidopsis* seedlings, temperature stress treatments were carried out as follows: WT and OE seedlings were planted in 1/2 MS plate at 24 °C under 16/8 h (light/dark) conditions with 75% relative humidity and 200 mmol·m^−2^·s^−1^ light intensity. For low-temperature stress, 2-week-old seedlings were transferred to 4 °C for 48 h. For high-temperature stress, the plants were transferred to 40 °C for 48 h. Two-week-old seedlings were sampled to analyze transcript level and physiological parameters. The 2 temperature stresses were performed in triplicate. T3 of *Arabidopsis* transgenic plants was analyzed.

Glutathione content and Fd-GOGAT enzyme activity were estimated according to previous studies [74,75]. MDA content and electrolyte leakage were measured with the methods of References [76,77]. Proline content was estimated by using the acid-ninhydrin method of Reference [78]. Hydrogen peroxide (H_2_O_2_) content and superoxide anion (O_2_^•−^) production rate were assayed with methods according to References [79,80]. Histochemical staining of H_2_O_2_ and O_2_^•−^ in leaves was carried out with 3,3-diaminobenzidine (DAB) and nitro blue tetrazolium (NBT) respectively, by the method of Reference [81].

### 4.10. Statistical Analysis

Values of physiology and biochemistry are expressed as means ± SE of 3 replications. Statistical significance (*p* < 0.05) was determined by ANOVA with SAS software (SAS Institute, Cary, NC, USA), and means were separated using Duncan’s multiple range test.

## 5. Conclusions

**Figure 10:**
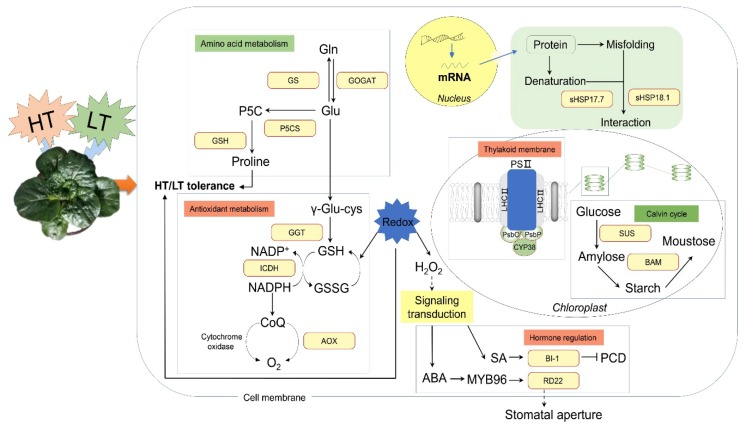
Schematic diagram of WS-1 proteins that were upregulated in response to both LT and HT. Red border represents upregulated proteins, arrows indicate positive regulation, and T-bars indicate negative regulation.

In this study, we identified 172 proteins that were upregulated in response to both LT and HT stress. Proteomic analysis showed that the temperature tolerance of WS-1 may be related to several factors, including steady redox homeostasis, an intact oxygen-evolving complex, the accumulation of osmolytes, the induction of sHSPs and chaperones, and enhanced metabolic processes. We infer that tolerance to LT and HT stress involves a common regulatory pathway, and that increased expression of DEPs, including Fe-GOGAT, BADH, P5CS, GGT, and AOX, helps regulate redox homeostasis (Figure 10). To further confirm the effect of redox homeostasis, we overexpressed *BccrGLU1* in *Arabidopsis*. Under LT and HT stress, *35S:BccrGLU1* plants exhibited higher GSH content and GSH/GSSG ratio and less oxidative damage compared to WT. These results are in agreement with the data of our comparative proteomics in WS-1. Combined with these data, redox homeostasis apparently played an important role in enhancing LT and HT stress tolerance in WS-1.

## Figures and Tables

**Table 1:** Primers used for fragment amplification of differentially expressed genes.

Accession	Name	Forward Primer (5′→3′)	Reverse Primer (5′→3′)
XM_009106490.1	*ALDH10A8*	TCGTCAATCCAGCAACCCAA	TCAGTCACCTTAGCGGCAAT
XM_013806620.2	*AOX1A*	AGCCATCTCTTGAAACCTGC	AGCGATTCCTTTGTTACCTCC
XM_009151277.2	*ERD10*	ACTGTTTGACTTCTTGG	GAGGAGAGTAGGCTTATG
XM_022705499.1	*RD22*	CAAACACTCCCATACCA	TACACCTCCCTTTCCAA
XM_013841006.2	*PSBP1*	TTTCACTCTCCAAACCCGTCCA	AGCTTCACCATAGGCGGCATC
XM_013830831.2	*PSBO1*	CAACCTCTGCTCTCGTCGTC	CTTGCTAACACTCTCGGCCT
XM_009129212.2	*ICDH*	AGTGAGGGAGGCTATGTGTG	CTATGCTGTTTGTGCTGGT
XM_009122518.1	*SUS1*	ATTTCATCATCACCAG	AGTCAATCTACGCTTC
XM_009118014.2	*P5CSB*	CCTTTTCCACCAAGATGCAC	CCCAGGCTTCATAACTAAACGA
XR_002653900.1	*CYP38*	CGGGAACTTTGTGGACTTGG	GCGTTTTCTTCCCAGTCACC
XM_002873500.2	*sHSP17.7*	AAGACCCGTAACAACCCT	CTTTTTCCACTCACCACA
XM_013858088.2	*sHSP18.1*	CAGCATTCACAAACGC	CCTCCTCATAAACTTC
XM_009127097.2	*Actin*	TGGGTTTGCTGGTGACGAT	TGCCTAGGACGACCAACAATACT

## Supplementary Materials

Supplementary materials can be found at https://www.mdpi.com/1422-0067/20/15/3760/s1.

## Abbreviations

DAB	3,3-diaminobenzidine
H_2_O_2_	Hydrogen peroxide
HT	High temperature
LC-MS/MS	Liquid chromatography–mass spectrometry
LT	Low temperature
MDA	Malondialdehyde
NBT	Nitro blue tetrazolium
NHCC	Non-heading Chinese cabbage
O_2_^•−^	Superoxide anion
ROS	Reactive oxygen species
TMT	Tandem mass tag
